# Identifying typologies of diurnal patterns in desk-based workers’ sedentary time

**DOI:** 10.1371/journal.pone.0248304

**Published:** 2021-04-09

**Authors:** Sayaka Kurosawa, Ai Shibata, Kaori Ishii, Mohammad Javad Koohsari, Koichiro Oka

**Affiliations:** 1 Graduate School of Sport Sciences, Waseda University, Tokorozawa, Saitama, Japan; 2 Faculty of Health and Sport Sciences, University of Tsukuba, Tsukuba, Ibaraki, Japan; 3 Faculty of Sport Sciences, Waseda University, Tokorozawa, Saitama, Japan; 4 Behavioural Epidemiology Laboratory, Baker Heart and Diabetes Institute, Melbourne, Victoria, Australia; 5 Melbourne School of Population and Global Health, The University of Melbourne, Melbourne, Victoria, Australia; Indiana University, UNITED STATES

## Abstract

The purpose of this study was to identify typologies of diurnal sedentary behavior patterns and sociodemographic characteristics of desk-based workers. The sedentary time of 229 desk-based workers was measured using accelerometer devices. The within individual diurnal variations in sedentary time was calculated for both workdays and non-workdays. Diurnal variations in sedentary time during each time period (morning, afternoon, and evening) was calculated as the percentage of sedentary time during each time period divided by the percentage of the total sedentary time. A hierarchical cluster analysis (Ward’s method) was used to identify the optimal number of clusters. To refine the initial clusters, a non-hierarchical cluster analysis (k-means method) was performed. Four clusters were identified: stable sedentary cluster (46.7%), off-morning break cluster (26.6%), off-afternoon break cluster (8.3%), and evening sedentary cluster (18.3%). The stable sedentary cluster had the lowest variations in sedentary time throughout the day and the highest amount of total sedentary time. Participants in the off-morning and off-afternoon break clusters had nearly the same sedentary patterns but took short-term breaks during non-workday mornings or afternoons. The evening sedentary cluster had a completely different pattern, with a longer sedentary time during the evening both on workdays and non-workdays. Sociodemographic attributes such as sex, household income, educational attainment, employment status, sleep duration, and residential area, differed significantly between groups. Initiatives to address desk-based workers’ sedentary behavior need to focus not only on the workplace but also on the appropriate timing for reducing excessive sedentary time in non-work contexts depending on the characteristics and diurnal patterns of target subgroups.

## Introduction

Sedentary behavior, defined as sitting or reclining, is characterized by an energy expenditure ≤1.5 metabolic equivalents (METs), and is a known health risk [1, 2]. For example, the longer time spent in sedentary behavior, which is distinct from not engaging in sufficient physical activity, increases health risks [1, 2]. Such health risks include all-cause and cardiovascular mortality, with some evidence that these occur in a dose-response manner [1, 2]. Highly sedentary at-risk populations include desk-based or office workers. Previous studies have shown that desk-based workers typically sit for approximately 70% of their workday [3]. This time is much higher compared to those of workers in other occupations [3] or for general middle-aged adults [4]. Even on non-working days, workers with higher occupational sedentary time have been reported to be more sedentary than those with more physically active job types [5]. Thus, it is prioritized to effectively intervene and reduce the overall levels of sedentary behavior among office workers.

Addressing more tailored messages and approaches to reducing sedentary behavior for targeted people maximizes the effectiveness of interventions for reducing sedentary time. This tailored approach may require audience segmentation, a process by which a large heterogeneous population is divided into a smaller number of homogeneous subgroups based on shared or similar characteristics/pattern of behavior [6]. When subgroups are identified and understood (e.g., by sociodemographic characteristics or other multilevel correlates), a more unique and specific approach that meets their needs and characteristics could be made available. In addition, such a cluster or typology may provide opportunity for future analyses to assess whether specific patterns of sedentary behavior are associated with health risks and even work performance.

A limited number of studies have identified unique typologies of individuals that distinguish distinct sedentary patterns, including clusters of screen time behavior in adolescents [7], physical activity and sedentary behavior in adolescents [8] and children [9], and domain-based sedentary behavior in older adults [10]. However, there is no study, according to our knowledge, conducted on the typology of patterns of sedentary behavior among office workers. In addition, previous studies examining the sedentary pattern among office workers have only focused on total sedentary behavior on workdays (including working hours and non-working hours) and non-workday frameworks [11]. Although office workers can routinely spend time in multiple sedentary behaviors on both work and non-work time with patterns differing by occupational status, domestic and social roles, pursuits, and preferences; their sedentary patterns can be influenced by circadian patterns or the time period of the day (e.g., morning, noon, and evening) [12, 13]. Previous studies have revealed that the amounts of sedentary behavior differ between morning and evening, even though these hours mostly appear to be categorized as non-working time [14, 15]. Thus, identifying and understanding behavioral typology that holds similar diurnal pattern of sedentary behavior may also be a meaningful way to provide more tailored and unique intervention strategies for specific subgroups of the office worker populations.

Therefore, this present study aimed to contribute to the limited research on desk-based workers’ sedentary behavior patterns across time-dependent contexts, by exploring: (1) whether desk-based workers form identifiable sedentary behavior clusters across the day time periods (morning, afternoon, evening), and (2) the total time spent in sedentary behavior and sociodemographic differences between members of different sedentary behavior clusters.

## Materials and methods

### Participants and procedure

**Fig 1:**
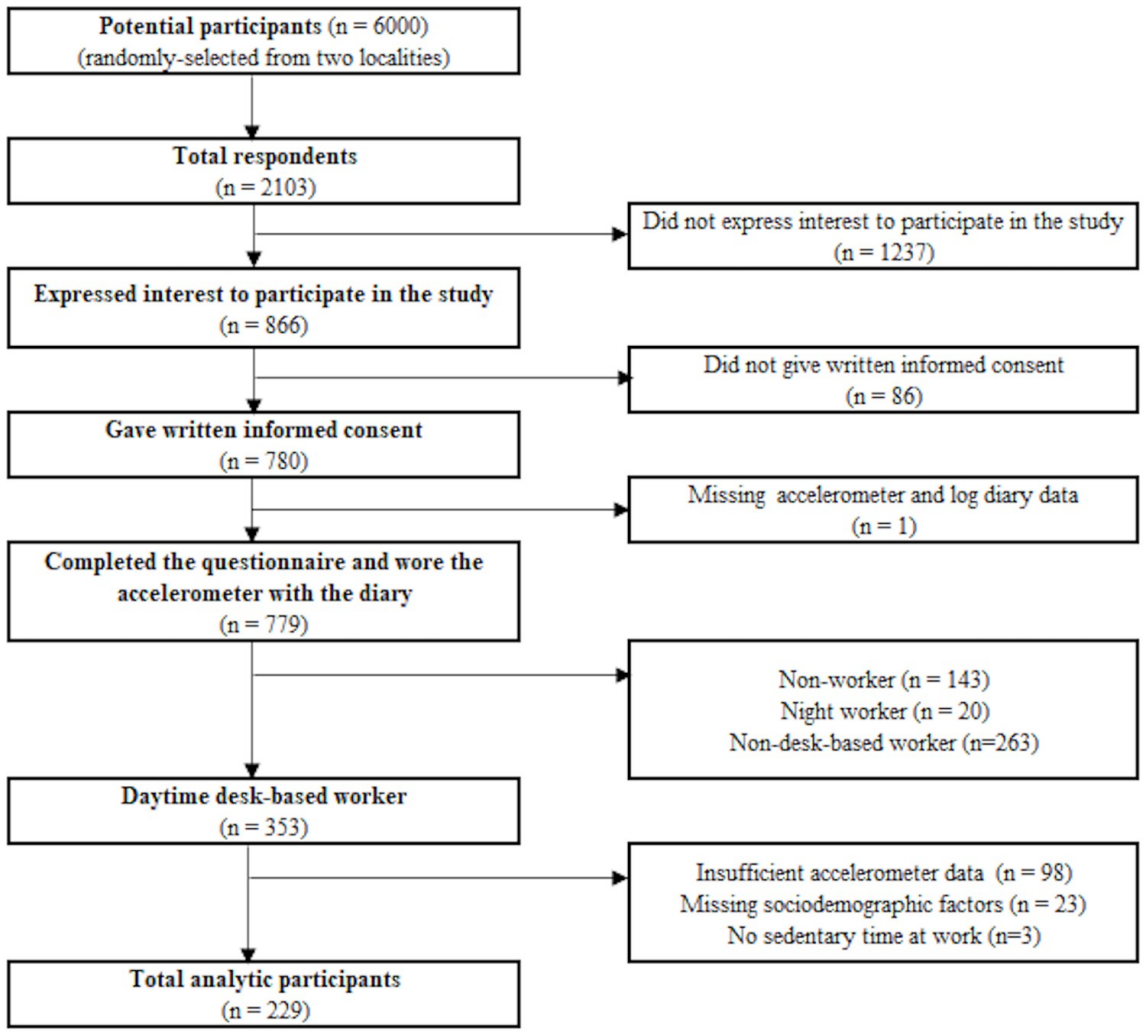
The flowchart of participant recruitment.

This cross-sectional survey was conducted from July to December 2013 and from April 2014 to February 2015, as part of a project to analyze social and urban design correlates of sedentary behaviors and physical activity among a sample of middle-aged adults in Japan. A total of 6,000 potential participants, aged 40–64 years, in two Japanese cities (Koto Ward: Tokyo metropolitan area; Matsuyama City: a local city of Shikoku region), were randomly selected from government residential registries, were stratified by sex and age (40–44, 45–49, 50–54, 55–59, and 60–64 years). Invitation letters were sent to all the potential participants. A reminder letter was sent to non-respondents. Those who expressed interest were mailed the informed consent form, the self-reported questionnaires that included sociodemographic and behavioral characteristics, and an accelerometer with a log diary. Those who signed the informed consent form, and completed and returned the questionnaire and accelerometer (with log diary) were 779 participants. A 1,000-yen (equivalent to about USD10) book voucher was offered to the participants who completed the questionnaire and wore the accelerometer with the diary. Daytime desk-based workers were included in this study (n = 353). Those who had insufficient accelerometer data (n = 98), no self-reported sedentary time in the domain of work in the questionnaire (n = 3), or missing or invalid data for the potential sociodemographic correlates (n = 23), were excluded. All the participants included in this analysis provided written informed consent. A flowchart of participant recruitment is shown in Fig 1. This study was approved by the Ethics Committee of Waseda University, Japan (number: 2012–269).

### Measurements

#### Objectively measured sedentary behavior and physical activity

Sedentary behavior and physical activity were objectively measured using a triaxial accelerometer (Active style Pro HJA-350IT; Omron Healthcare Co. Ltd., Kyoto, Japan). The accelerometer estimated the metabolic equivalents every 60-sec epoch, based on the tri-axial accelerations. High validity and reliability were previously reported for the METs, estimated by this accelerometer [16, 17]. The accelerometer used in this study recorded the anteroposterior (x-axis), mediolateral (y-axis), and vertical (z-axis) accelerations with a resolution of 3 mG at 32 Hz. This type of accelerometer directly predicts the metabolic equivalents without the need for any additional process, using a multiple regression model, which is based on 12 key activities (seven locomotive and five household activities) [16]. The participants were instructed to wear the accelerometer on the left side of the waist during waking hours for seven consecutive days and to remove it during water-based activities and contact sports.

Data management of the accelerometer data was processed using the Omron Health Management Software, BI-LINK for physical activity professional edition V.1.0 and custom software. Non-wear accelerometer time was defined as intervals of at least 60 consecutive minutes of no activity (0.9 or less METs) [16], with an allowance of up to two minutes of observations for some limited movements (≤ 1.0 METs) within these periods [18, 19]. Due to sparse data in the early morning and late evening, behavioral measures were calculated between 06:00–23:59 and each time period of the day [morning (06:00–11:59), afternoon (12:00–17:59), and evening (18:00–23:59)] on workdays and non-workdays.

Days with at least 10 hours/day of wear time were regarded as valid [18, 19]. The periods of wearing the accelerometer and days of the week were verified by the log diaries and checked against the accelerometer data. To estimate workday and non-workday patterns, participants who completed four or more valid days of data with at least three workdays and a non-workday were included. Because of noticeable non-wear accelerometer time in the morning and evening, those who had a minimum of 25% valid hours of wearing time in each of the three time periods [20] of at least three workdays and a non-workday were eligible for this study [11], in order to ensure the representativeness of accelerometer data during each time period, on workdays and non-workdays.

Two measures of sedentary behavior were expressed for each time period (morning, afternoon, evening) on workdays and non-workdays: sedentary time (minutes/period) and percentage of sedentary time in wear-time (%). Additionally, sedentary behavior and physical activity were expressed for the total time periods as follows: sedentary, light-intensity physical activity (LIPA), and moderate-to-vigorous intensity physical activity (MVPA) time (minutes/day); percentage of sedentary, LIPA, and MVPA time in wear time (%); and number of sedentary breaks (times/sedentary hour). Sedentary behavior, LIPA, and MVPA were defined as an accelerometer-estimated intensity of ≤ 1.5 METs, > 1.5 to < 3.0 METs, and 3.0 or more METs, respectively [21]. A sedentary break was defined as a period of non-sedentary bouts between two sedentary bouts [22].

#### Self-reported sedentary behavior in different domains

Participants reported the daily average time spent in sedentary behavior for six domains: while riding in a car as a driver or passenger; using public transport; at work; watching television, videos, and digital video discs; using a computer, cell phone, and tablet personal computer for non-work purposes; and sitting for other purposes in leisure time (e.g., talking, reading, listening to music, or engaging in a hobby). They were asked to provide a separate response for workdays and non-workdays over the past seven days. This scale has a fair to good validity for estimating total sedentary time against an objective measurement, using an accelerometer, and high reliability, among middle-aged adults in Japan [23].

#### Potential sociodemographic variables

Sex and age were obtained from the government residential registries. Marital status (currently single, married), household income (< 5 million yen, ≥ 5 million yen), educational attainment (high school or less, two years of college or higher education), employment status (full-time, part-time), days of the week for non-workday (weekend, ≥ one day from weekday), height, weight, smoking status (smoker, nonsmoker), alcohol consumption (≤ 1–3 times/month, ≥ once a week), sleep duration (< 6 hours/day, ≥ 6 hours/day), and car ownership (yes, no) were self-reported in the questionnaire. The Global Physical Activity Questionnaire (GPAQ) was utilized to estimate the habitual time spent in MVPA during leisure time (0 min/day, > 0 min/day). Body mass index (BMI; kg/m2) was calculated from self-reported height and weight, and defined as normal weight (< 25 kg/m2) and overweight (≥ 25 kg/m2). The residential area was categorized as urban (Koto) or suburban (Matsuyama).

### Statistical analysis

Participants were clustered based on the similarity in their objectively measured sedentary behavior patterns for each time period: workday morning, workday afternoon, workday evening, non-workday morning, non-workday afternoon, and non-workday evening. First, the within individual diurnal variation in sedentary time (i.e., ‘variation level’) was calculated for workday and non-workday, respectively. Sedentary time (minutes/period) could be subject to participant-specific non-wear patterns, especially in the morning and afternoon (due to differences in waking up or going to sleep times). Taking this into consideration, the sedentary time variation level during each time period was based on the time period/whole day ratio, calculated as the percentage of sedentary time (% of wear time) during each time period divided by the percentage of the total sedentary time for the whole day (% of wear time). A two-step clustering procedure was performed, based on the sedentary time variation level, during each time period. First, a dendrogram was generated through agglomerative hierarchical clustering (Ward’s method) to identify the optimal number of clusters. Ward’s method tends to derive more equally sized groups, and four clusters emerged. Subsequently, non-hierarchical, k-means partitioning cluster analysis was performed to create four clusters, aiming to refine the initial cluster and to reduce the risk of cluster misassignment, common with hierarchical clustering methods.

One-way analysis of variance and post hoc Bonferroni multiple comparison tests for continuous variables and χ^2^ tests and post hoc residual analyses for categorical variables were used to identify differences in sociodemographic variables across the clusters. In order to examine differences in the average values of sedentary behavior and physical activity during each time period (whole day, morning, afternoon, evening) and domain-specific sedentary behavior among the four divided clusters, analysis of variance tests and post hoc Bonferroni multiple comparison tests were used. In addition to the percentage of sedentary time in each cluster, to understand by how much the sedentary time accumulated during each time period of the day, is an important information to determine the potential timing required to reduce sedentary behavior. Therefore, differences in time spent in sedentary behavior (minutes/period) and the percentage of sedentary time in wear-time (%) between the four clusters, were examined. Participants who reported missing or invalid data in domain-specific sedentary time were excluded from the analysis of time spent in the sedentary behavior of each domain. Statistical analyses were performed using IBM SPSS Statistics for Windows, version 26.0 (IBM Japan Inc., Tokyo, Japan). The level of significance was set at p<0.05.

## Results

Overall, 229 participants with a mean age of 51.0 (standard deviation [SD] = 6.8) years, were included in this analysis. Of the 229, 47.6% were women, 67.7% had a household income ≥ 5 million yen, 73.4% had graduated with two years of college or higher education, and 87.8% were full-time workers.

**Fig 2:**
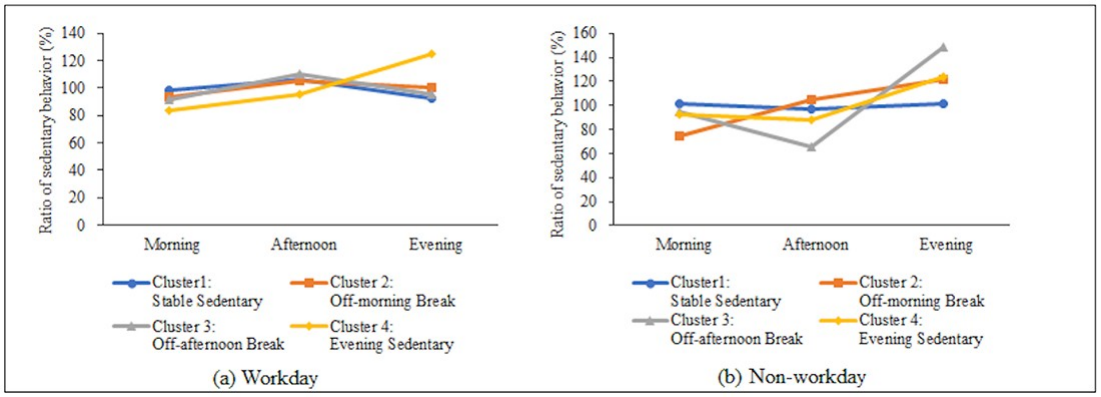
Diurnal patterns of sedentary behavior in four clusters. Variation level of sedentary time during each time period was based on the time period/whole day ratio. (a) Variation level of the workday was calculated as the percentage of sedentary time during each time period on workdays, divided by the percentage of the total sedentary time on the whole workday. (b) Variation level of the non-workday was calculated as the percentage of sedentary time during each time period on non-workdays divided by the percentage of total sedentary time on the whole non-workday.

The final result of the cluster analyses for the 229 participants who completed the measurements, is presented in Fig 2. Four distinct clusters emerged. The cluster names were based on the individual patterns of objectively measured sedentary behavior, over the three time periods, for each workday and non-workday.

Cluster 1 (n = 107, 46.7%) had a low fluctuation pattern across the day and has therefore been termed ‘Stable Sedentary’. The mean (SD) variation level of sedentary behavior among this cluster ranged from 92.9 (12.8)% (evening) to 106.1 (8.0)% (afternoon) on workday, and 97.4 (12.1)% (afternoon) to 101.7 (12.9)% (evening) on non-workdays. Cluster 2 (n = 61, 26.6%) had a flat pattern most of the time, but had a dip on the non-workday mornings, and was termed ‘Off-morning Break’. The mean variation level of sedentary behavior among this cluster ranged from 93.5 (10.1)% (morning) to 105.4 (10.0)% (afternoon) on workdays, and 74.1 (11.1)% (morning) to 121.5 (13.4)% (evening) on non-workdays. Cluster 3 (n = 19, 8.3%) had a flat pattern most of the time, but had a dip on the non-workday afternoons, and was termed ‘Off-afternoon Break’. The mean variation level of sedentary behavior among this cluster ranged from 91.4 (7.5)% (morning) to 109.8 (7.7)% (afternoon) on workdays, and 66.1 (15.4)% (afternoon) to 149.0 (25.8)% (evening) on non-workdays. Cluster 4 (n = 42, 18.3%) had a spike of sedentary evening times, whereas sedentary times in the mornings and afternoons were lower, and was termed ‘Evening Sedentary’. The mean variation level of sedentary behavior among this cluster ranged from 83.2 (11.3)% (morning) to 125.2 (15.1)% (evening) on workdays, and 87.7 (9.1)% (afternoon) to 124.3 (17.4)% (evening) on non-workdays. The patterns of variation level for each cluster were similar to the patterns of the absolute percentage of sedentary time (% wear time) in the corresponding time period for each cluster (S1 Table).

Table 1 presents the characteristics of the participants by cluster. Those of stable sedentary cluster were more likely to have higher educational attainment, full-time work, and shorter sleep duration than those of other clusters. Those of off-morning break cluster were more likely to have longer sleep duration than those of other clusters. Those of off-afternoon break cluster were more likely to have higher household income and educational attainment and to live in the urban residential area (Koto) than those of other clusters. Those of evening sedentary cluster were more likely to be women, to have lower household income, lower educational attainment, part-time work, low frequency of alcohol consumption, longer sleep duration, and living in the suburban residential area (Matsuyama) than those of other clusters.

**Table 1:** Characteristics of participants in four clusters.

	Total (n = 229)		Cluster 1: Stable Sedentary (n = 107,46.7%)		Cluster 2: Off-morning Break (n = 61, 26.6%)		Cluster 3: Off-afternoon Break (n = 19, 8.3%)		Cluster 4: Evening Sedentary (n = 42, 18.3%)		p
	n	%	n	%	n	%	n	%	n	%	
Age, years (mean, SD)	51.0	6.8	50.5	6.6	51.3	6.4	53.4	7.1	51.0	7.8	0.417
Sex: women	109	47.6	47	43.9	24	39.3	9	47.4	29	69.0*	0.019
Marital status: married	176	76.9	81	75.7	49	80.3	14	73.7	32	76.2	0.894
Household income											
< 5 million yen	74	32.3	37	34.6	17	27.9	1	5.3*	19	45.2*	0.016
≥ 5 million yen	155	67.7	70	65.4	44	72.1	18	94.7	23	54.8	
Educational attainment											
≤ High school	61	26.6	20	18.7*	20	32.8	1	5.3*	20	47.6*	<0.001
≥ Two years of college	168	73.4	87	81.3	41	67.2	18	94.7	22	52.4	
Employment status											
Full-time	201	87.8	99	92.5*	53	86.9	18	94.7	31	73.8*	0.013
Part-time	28	12.2	8	7.5	8	13.1	1	5.3	11	26.2	
Days of week for non-workday:	66	28.8	32	29.9	19	31.1	5	26.3	10	23.8	0.851
≥ 1 day from weekday											
Body mass index, kg/m^2^											
< 25	178	77.7	77	72.0	47	77.0	15	78.9	39	92.9	0.054
≥ 25	51	22.3	30	28.0	14	23.0	4	21.1	3	7.1	
Smoking status: smokers	33	14.4	15	14.0	6	9.8	4	21.1	8	19.0	0.483
Alcohol consumption											
≤ 1–3 times/month	109	47.6	51	47.7	23	37.7	7	36.8	28	66.7*	0.024
≥ Once a week	120	52.4	56	52.3	38	62.3	12	63.2	14	33.3	
Sleep duration											
< 6 hours/day	108	47.2	65	60.7*	22	36.1*	7	36.8	14	33.3*	0.002
≥ 6 hours/day	121	52.8	42	39.3	39	63.9	12	63.2	28	66.7	
MVPA habit in leisure time:	113	49.3	55	51.4	30	49.2	12	63.2	16	38.1	0.289
> 0 min/day											
Car ownership: Yes	163	71.2	69	64.5	46	75.4	14	73.7	34	81.1	0.181
Residential area											
Matsuyama	90	39.3	39	36.4	25	41.0	3	15.8*	23	54.8*	0.029
Koto	139	60.7	68	63.6	36	59.0	16	84.2	19	45.2	

MVPA, moderate-to-vigorous intensity physical activity; SD, standard deviation

Using analysis of variance with post hoc Bonferroni multiple comparison tests for continuous value and χ2 tests with post hoc residual analyses for categorical values, group differences were examined.

* Adjusted standardized residual > 1.96

The total time spent in sedentary behavior for the whole day differed by cluster group (Table 2). Overall, the stable sedentary cluster engaged in a significantly higher percentage of sedentary behavior (64.8%) than the off-morning break cluster (60.3%) and evening sedentary cluster (56.8%). On workdays, the stable sedentary cluster (65.1%), off-morning break cluster (62.4%), and off-afternoon break cluster (64.0%) engaged in a significantly higher percentage of sedentary time than the evening sedentary cluster (55.8%). The stable sedentary cluster also spent a significantly higher percentage of sedentary time (64.2%) than the off-morning break cluster (55.2%) and off-afternoon break cluster (47.4%) on non-workdays. In addition, the off-afternoon break cluster spent a significantly lower percentage of sedentary time than the evening sedentary cluster on non-workdays (59.1%). Compared with the patterns of sedentary behavior, almost all the inverse differences were shown for the sedentary break and LIPA. Although there was no difference in overall and workday MVPA, a significantly higher percentage of time engaged in MVPA on non-workdays was shown among the off-afternoon break cluster (9.8%; 87.9 minutes/day) than in the stable sedentary cluster (5.8%; 49.6 minutes/day) and evening sedentary cluster (5.5%; 48.1 minutes/day).

**Table 2:** Differences in the total amounts of objectively-measured sedentary behavior and physical activity by cluster.

	Cluster 1: Stable Sedentary (n = 107,46.7%)		Cluster 2: Off-morning Break (n = 61, 26.6%)		Cluster 3: Off-afternoon Break (n = 19, 8.3%)		Cluster 4: Evening Sedentary (n = 42, 18.3%)		p	Post hoc
n = 229	Mean	SD	Mean	SD	Mean	SD	Mean	SD		
Wear time (min)										
Workday	929.7	76.3	947.6	63.1	909.3	75.7	951.4	65.0	0.071	
Non-workday	864.9	102.1	888.3	85.3	900.6	74.5	880.3	62.7	0.234	
Overall	911.2	73.1	930.7	57.9	906.8	69.6	931.1	53.7	0.144	
SB (min)										
Workday	603.8	96.8	590.0	98.6	578.0	63.9	531.0	83.0	<0.001	1>4,2>4
Non-workday	554.9	128.0	491.0	121.2	426.3	107.0	520.6	135.5	<0.001	1>2,1>3,3<4
Overall	589.8	92.9	561.7	92.5	534.7	66.1	528.1	83.9	0.001	1>4
SB (%)										
Workday	65.1	9.9	62.4	10.6	64.0	9.2	55.8	8.1	<0.001	1>4,2>4,3>4
Non-workday	64.2	12.9	55.2	12.3	47.4	11.3	59.1	15.1	<0.001	1>2,1>3,3<4
Overall	64.8	9.6	60.3	9.9	59.3	8.5	56.8	8.8	<0.001	1>2,1>4
Break (times/sedentary hour)										
Workday	8.2	2.8	8.7	3.1	8.3	2.6	10.5	2.6	<0.001	1<4,2<4,3<4
Non-workday	7.4	3.2	9.4	3.9	10.6	2.8	8.6	4.0	<0.001	1<2,1<3
Overall	8.0	2.6	8.9	2.8	8.9	2.4	10.0	2.5	0.001	1<4
LIPA (%)										
Workday	28.0	9.4	30.7	9.9	28.5	8.9	37.9	7.5	<0.001	1<4,2<4,3<4
Non-workday	30.0	11.3	37.7	10.7	42.8	9.6	35.4	13.0	<0.001	1<2,1<3
Overall	28.6	8.9	32.7	9.0	32.6	8.0	37.2	7.7	<0.001	1<2,1<4
MVPA (%)										
Workday	6.9	3.5	6.9	2.8	7.4	3.3	6.3	2.8	0.557	
Non-workday	5.8	3.7	7.1	4.0	9.8	4.9	5.5	3.4	<0.001	1<3,3>4
Overall	6.6	3.1	7.0	2.8	8.1	3.3	6.0	2.7	0.074	

SB, sedentary behavior; LIPA, light-intensity physical activity; MVPA, moderate-to-vigorous intensity physical activity; SD, standard deviation.

Using analysis of variance with post hoc Bonferroni multiple comparison tests, group differences were examined.

Table 3 shows the self-reported time spent in sedentary behavior for each domain of the four clusters. On a workday, stable sedentary cluster had significantly more minutes of sedentary behavior during public transport than evening sedentary cluster. Evening sedentary cluster had significantly longer sedentary time for TV viewing on a workday than off-morning break cluster. On non-workday, off-afternoon break cluster significantly spent more minutes of sedentary behavior in the car than stable sedentary cluster. Off-afternoon break cluster also significantly spent more minutes of sedentary behavior on public transport than all other clusters.

**Table 3:** Differences in domain-specific sedentary behavior by clusters.

	Cluster 1: Stable Sedentary (n = 107,46.7%)		Cluster 2: Off-morning Break (n = 61, 26.6%)		Cluster 3: Off-afternoon Break (n = 19, 8.3%)		Cluster 4: Evening Sedentary (n = 42, 18.3%)		p	Post hoc
	Mean	SD	Mean	SD	Mean	SD	Mean	SD		
**Domain-specific SB (min/day)**										
Workday										
Car (n = 200)	22.8	46.8	14.9	30.6	11.9	28.7	25.1	36.3	0.484	
Public transport (n = 215)	29.3	42.7	16.9	29.5	34.7	44.0	9.2	17.5	0.007	1>4
Work (n = 223)	436.4	123.3	417.6	137.4	423.2	144.3	376.7	128.3	0.105	
TV (n = 223)	115.1	73.5	101.1	70.2	96.3	44.2	145.5	92.7	0.019	2<4
PC (n = 223)	56.9	67.8	40.1	46.0	78.2	104.3	71.3	84.1	0.077	
Other leisure (n = 220)	39.7	31.2	39.1	31.6	33.4	18.1	39.0	27.2	0.870	
Non-workday										
Car (n = 201)	30.5	37.7	35.6	41.0	66.0	70.1	42.9	49.3	0.027	1<3
Public transport (n = 206)	6.6	18.6	6.8	16.0	58.5	121.4	7.8	19.3	<0.001	1<3,2<3,3>4
Work (n = 200)	34.7	95.1	29.4	97.5	75.0	178.7	22.9	76.9	0.408	
TV (n = 220)	242.7	153.6	193.4	119.0	195.8	95.1	210.4	146.8	0.137	
PC (n = 219)	81.7	107.4	68.3	70.5	62.8	55.8	98.9	133.1	0.435	
Other leisure (n = 219)	79.9	70.6	66.5	57.4	69.5	35.5	78.5	74.8	0.615	

SB, sedentary behavior; SD, standard deviation; PC, personal computer; TV, television.

Using analysis of variance with post hoc Bonferroni multiple comparison tests, group differences were examined.

## Discussion

Segmentation of the populations based on their shared behavioral characteristics is an essential first step towards developing more effective and persuasive intervention strategies than a one-size-fits-all strategies. This is the first study, to our knowledge, to identify the typologies of objectively-measured diurnal sedentary behavior patterns using a clustering approach among desk-based workers. Four profiles, including the ‘Stable Sedentary,’ ‘Off-morning Break,’ ‘Off-afternoon Break,’ and ‘Evening Sedentary’ groups, were identified. Additionally, about half of the office-based workers in our sample had a shared stable sedentary pattern throughout the day and week (stable sedentary group). Also, this group spent approximately 65% of the day in sedentary, which was 4–8% higher than for those in the other three groups. Given that prolonged sedentary behavior has been reported to be adversely associated not only with health outcomes but also with engagement and productivity among workers [24], developing intervention strategies centered on the characteristics of those with a stable sedentary pattern may maximize the worksite intervention effects, to reduce sedentary behavior.

Except for the desk-based workers in the evening sedentary group, there was little difference in the diurnal sedentary patterns among the other three groups (stable sedentary, off-morning break, and off-afternoon break groups) with > 80% of the desk-based workers’ remaining sedentary throughout the workday. On the other hand, non-workday patterns of sedentary behavior, the proportion of time spent in LIPA, and MVPA among these classes were slightly different. Previous studies have reported that diurnal routines of activities such as travel, personal care, TV, and leisure varied among adults [25]. Our findings confirmed that the desk-based workstyle, which is commonly determined by occupational demands, can have the most significant impact on whole-day routines and patterns of sedentary behavior on workdays [3, 13, 26]. In contrast, the opportunities and timing for breaking up sedentary behavior, and the intensity of physical activity accompanying the breaking up sedentary behavior on non-workdays may vary depending on individuals domestic and leisure characteristics or status. The present findings imply that workplace intervention strategies for reducing sitting time at work, such as organizational and environmental changes (e.g., providing adjustable height desks to enable sitting or standing at work, policy changes, or computer prompts) may be useful for a wide range of desk-based workers, regardless of the difference in non-working diurnal sedentary patterns. In addition, counseling and information provision tailored towards each of the three groups’ non-workday diurnal patterns of sedentary behavior, may expand the effectiveness of workplace intervention to reduce leisure-time sitting time. Moreover, since a quarter of desk-based workers have entirely different patterns of sedentary behavior even on a workday, worksite intervention may need to consider the patterns and contexts of sedentary behavior other than those at working hours.

The current study found that sociodemographic characteristics such as sex, household income, educational attainment, employment status, alcohol consumption, sleep duration, and residential area differed by group. In addition, group differences were found in domain-specific sedentary behavior based on car use, public transport use, and TV watching. It is possible that the diurnal patterns of sedentary behavior distinguishing the four aforementioned groups may be influenced by intrapersonal correlates (e.g., demographics and behavioral correlates) as well as social and environmental differences. Socioeconomic status (e.g., educational attainment, occupation, and household income) and environment (e.g., home and neighborhood environment), also identified as correlates of sedentary behavior in the previous studies [27], are partly but closely related to the residential area. However, this study did not directly examine occupation. Further study would need to consider more specific work-related attributes, such as occupation and job position. To develop a tailored approach addressing sedentary behavior, future studies should present more evidence on the modifiable correlates of sedentary behavior in each group.

The present study has several limitations. First, we were unable to determine causal relationships between variables because of the cross-sectional design of this study. Second, the types of accelerometer devices used in this study have limitations in measuring water-based activities, movement of the legs, and arm activities. They also cannot accurately distinguish between sitting and very-static standing postures. In addition, since the accelerometer wearing time varied among participants (due to waking up or going to sleep time differences), this study cannot refer fully to the absolute time spent in sedentary behavior during each time period. Lastly, the response rate was low in this study, and there may have been selection bias. Future studies are needed that can contribute to the intervention efforts based on more specific behavior patterns. The future study can evaluate the quantity of behavior based on an objective evaluation method while the quality of the contents of behavior based on a subjective evaluation method. This is the first study to report diurnal pattern typologies of sedentary behavior among desk-based workers. Another strength of this study was the objective measurement of sedentary behavior and physical activity.

## Conclusion

In summary, in this sample, there were four different clusters among the desk-based workers. This study found that a substantial proportion of desk-based workers (stable sedentary group) may be classified into patterns of behaviors that represent high levels of sedentary behavior throughout the workdays and non-workdays. Participants in the off-morning break and off-afternoon break groups had a nearly sedentary pattern but took a short-term break during non-workday mornings or afternoons. The rest (evening sedentary group) had a completely different pattern, and sedentary time was longer during the evenings on both workdays and non-workdays. Sociodemographic attributes such as sex, household income, educational attainment, employment status, sleep duration, and residential area, differed by group. Although initiatives to address desk-based workers’ sedentary behavior could focus on reducing sedentary behavior at the workplace, considering characteristics and diurnal patterns in nonworkplace may improve the effectiveness of interventions to reducing sedentary behavior.

## Supporting information

S1 Table Differences in time-specific sedentary behavior by cluster. SB, sedentary behavior; SD, standard deviation. Using analysis of variance with post hoc Bonferroni multiple comparison tests, group differences were examined.
(DOCX)
