# Supplementary material for: Identifying typologies of diurnal patterns in desk-based workers’ sedentary time
Source: PLoS One. 2021 Apr 9;16(4):e0248304. doi: 10.1371/journal.pone.0248304 (PMC8034739; doi:10.1371/journal.pone.0248304)
Supplement: S1 Table — SB, sedentary behavior; SD, standard deviation. Using analysis of variance with post hoc Bonferroni multiple comparison tests, group differences were examined. (DOCX) [file pone.0248304.s001.docx]

**S1 Table. Differences in time-specific sedentary behavior by cluster**

|  | **Cluster1:**  **Stable Sedentary**  **(n=107,46.7%)** | | **Cluster 2:**  **Off-morning Break**  **(n=61, 26.6%)** | | **Cluster 3:**  **Off-afternoon Break**  **(n=19, 8.3%)** | | **Cluster 4:**  **Evening Sedentary**  **(n=42, 18.3%)** | | **p** | **Post hoc** |
| --- | --- | --- | --- | --- | --- | --- | --- | --- | --- | --- |
| n=229 | Mean | SD | Mean | SD | Mean | SD | Mean | SD |  |  |
| ***Time-specific SB*** | |  |  |  |  |  |  |  |  |  |
| **SB (%)** |  |  |  |  |  |  |  |  |  |  |
| Workday |  |  |  |  |  |  |  |  |  |  |
| Morning | 63.8 | 10.2 | 58.5 | 12.0 | 58.6 | 10.0 | 46.8 | 10.2 | <0.001 | 1>2, 1>4, 2>4, 3>4 |
| Afternoon | 69.1 | 11.6 | 65.8 | 12.6 | 70.2 | 11.2 | 53.2 | 10.3 | <0.001 | 1>4,2>4,3>4 |
| Evening | 60.6 | 12.4 | 62.2 | 11.6 | 61.1 | 10.1 | 69.3 | 9.1 | 0.001 | 1<4,2<4 |
| Non-workday |  |  |  |  |  |  |  |  |  |  |
| Morning | 64.9 | 13.6 | 41.2 | 12.2 | 45.6 | 17.3 | 55.3 | 17.5 | <0.001 | 1>2,1>3,1>4,2<4 |
| Afternoon | 62.8 | 15.7 | 57.7 | 13.6 | 31.7 | 11.6 | 52.3 | 16.4 | <0.001 | 1>3,1>4,2>3,3<4 |
| Evening | 65.3 | 15.1 | 66.9 | 15.7 | 69.1 | 13.2 | 71.9 | 14.3 | 0.105 |  |
| **SB (min)** |  |  |  |  |  |  |  |  |  |  |
| Workday |  |  |  |  |  |  |  |  |  |  |
| Morning | 188.1 | 40.6 | 189.2 | 41.2 | 166.4 | 31.6 | 145.7 | 36.3 | <0.001 | 1>4,2>4 |
| Afternoon | 246.0 | 43.7 | 234.0 | 45.1 | 252.6 | 40.2 | 190.7 | 37.4 | <0.001 | 1>4,2>4,3>4 |
| Evening | 169.7 | 50.0 | 166.8 | 46.5 | 159.1 | 33.2 | 194.7 | 42.6 | 0.007 | 1<4,2<4,3<4 |
| Non-workday |  |  |  |  |  |  |  |  |  |  |
| Morning | 153.2 | 50.7 | 114.9 | 39.2 | 120.0 | 43.7 | 139.1 | 44.0 | <0.001 | 1>2,1>3 |
| Afternoon | 216.3 | 59.2 | 198.7 | 46.9 | 112.0 | 43.9 | 180.4 | 59.7 | <0.001 | 1>3,1>4,2>3,3<4 |
| Evening | 185.4 | 62.4 | 177.5 | 59.7 | 194.4 | 55.6 | 201.1 | 63.3 | 0.259 |  |
| **Wear time (min)** |  |  |  |  |  |  |  |  |  |  |
| Workday |  |  |  |  |  |  |  |  |  |  |
| Morning | 295.2 | 47.8 | 325.0 | 35.6 | 286.5 | 47.2 | 311.8 | 36.0 | <0.001 | 1<2,2>3 |
| Afternoon | 355.7 | 12.9 | 355.8 | 12.8 | 359.6 | 1.8 | 358.3 | 6.4 | 0.364 |  |
| Evening | 278.8 | 55.8 | 266.8 | 48.8 | 263.2 | 48.9 | 281.3 | 51.2 | 0.312 |  |
| Non-workday |  |  |  |  |  |  |  |  |  |  |
| Morning | 237.9 | 64.6 | 279.8 | 58.6 | 268.7 | 42.7 | 259.2 | 59.3 | <0.001 | 1<2 |
| Afternoon | 344.5 | 36.0 | 345.2 | 26.5 | 349.1 | 42.8 | 344.3 | 25.5 | 0.953 |  |
| Evening | 282.5 | 65.4 | 263.4 | 56.1 | 282.8 | 63.6 | 276.8 | 57.4 | 0.264 |  |

SB, sedentary behavior; SD, standard deviation.

Using analysis of variance with post hoc Bonferroni multiple comparison tests, group differences were examined.
